# Reporting on the Role of miRNAs and Affected Pathways on the Molecular Backbone of Ovarian Insufficiency: A Systematic Review and Critical Analysis Mapping of Future Research

**DOI:** 10.3389/fcell.2020.590106

**Published:** 2021-01-12

**Authors:** Anna Rapani, Dimitra Nikiforaki, Dimitra Karagkouni, Konstantinos Sfakianoudis, Petroula Tsioulou, Sokratis Grigoriadis, Evangelos Maziotis, Amelia Pantou, Aikaterini Voutsina, Agni Pantou, Michael Koutsilieris, Artemis Hatzigeorgiou, Konstantinos Pantos, Mara Simopoulou

**Affiliations:** ^1^Department of Physiology, Medical School, National and Kapodistrian University of Athens, Athens, Greece; ^2^Assisted Conception Unit, 2nd Department of Obstetrics and Gynecology, Aretaieion Hospital, Medical School, National and Kapodistrian University of Athens, Athens, Greece; ^3^DIANA-Lab, Department of Computer Science and Biomedical Informatics, University of Thessaly, Lamia, Greece; ^4^Hellenic Pasteur Institute, Athens, Greece; ^5^Centre for Human Reproduction, Genesis Athens Clinic, Athens, Greece

**Keywords:** ovarian insufficiency, poor ovarian response, premature ovarian failure/insufficiency, advanced maternal age, miRNAs, profiling

## Abstract

Ovarian insufficiency is identified as a perplexing entity in the long list of pathologies impairing fertility dynamics. The three distinct classifications of ovarian insufficiency are poor ovarian response, premature ovarian insufficiency/failure, and advanced maternal age, sharing the common denominator of deteriorated ovarian reserve. Despite efforts to define clear lines among the three, the vast heterogeneity and overlap of clinical characteristics renders their diagnosis and management challenging. Lack of a consensus has prompted an empirically based management coupled by uncertainty from the clinicians’ perspective. Profiling of patients in the era of precision medicine seems to be the way forward, while the necessity for a novel approach is underlined. Implicating miRNAs in the quest for patient profiling is promising in light of their fundamental role in cellular and gene expression regulation. To this end, the current study sets out to explore and compare the three pathophysiologies—from a molecular point of view—in order to enable profiling of patients in the context of *in vitro* fertilization treatment and enrich the data required to practice individualized medicine. Following a systematic investigation of literature, data referring to miRNAs were collected for each patient category based on five included studies. miRNA–target pairs were retrieved from the DIANA-TarBase repository and microT-CDS. Gene and miRNA annotations were derived from Ensembl and miRbase. A subsequent gene-set enrichment analysis of miRNA targets was performed for each category separately. A literature review on the most crucial of the detected pathways was performed to reveal their relevance to fertility deterioration. Results supported that all three pathophysiologies share a common ground regarding the affected pathways, naturally attributed to the common denominator of ovarian insufficiency. As evidenced, miRNAs could be employed to explore the fine lines and diverse nature of pathophysiology since they constitute invaluable biomarkers. Interestingly, it is the differentiation through miRNAs and not through the molecular affected pathways that corresponds to the three distinctive categories. Alarming discrepancies among publications were revealed, pertaining to employment of empirical and arbitrary criteria in categorizing the patients. Following bioinformatic analysis, the final step of the current study consisted of a critical analysis of the molecular data sourced, providing a clear and unique insight into the physiological mechanisms involved. It is our intention to contribute to mapping future research dedicated to ovarian insufficiency and to help researchers navigate the overwhelming information published in molecular studies.

## Introduction

Since its conceptualization—nearing five decades ago—assisted reproductive technologies (ART) emerged as a highly promising field in the 1970s as patients experienced an individualized approach. This tailor-made approach accounted for the variation on age, reproductive history, infertility etiology, and all contributing factors synthesizing the unique profile of each infertile couple. Therefore, even prior to introducing the concept of precision medicine, ART presented with a head start. Fast forward to today, one would expect ART to have progressed accordingly. Nonetheless, the majority of studies conducted on the field conclude that further individualization is required, especially for optimal management of certain highly demanding and perplexing pathologies entailed in infertility such as ovarian insufficiency.

Ovarian insufficiency refers to a state of compromised ovarian status regularly encountered as an infertility etiology within the ART context. A range of different classifications and diagnoses are included, namely, poor ovarian response (POR), premature ovarian failure/premature ovarian insufficiency (POF/POI), and advanced maternal age (AMA). The pathways leading to the aforementioned cases of ovarian insufficiency implicate a variety of iatrogenic, idiopathic, pathological, and physiological processes, acting singularly or in combination, but unmistakably leading to the diagnosis of ovarian insufficiency (Marder et al., 2012; Torrealday et al., 2017; Rudnicka et al., 2018; Ubaldi et al., 2019). At any rate, the common denominator for all types of ovarian insufficiency is infertility; therefore, common ground is shared and overlap in treatment is inevitable. The heterogeneity of patient characteristics, attributed to each pathology, results in severe discrepancies, serving as confounders or obstacles in diagnosing with precision and aptly identifying either category of ovarian insufficiency. The demanding nature of distinguishing between these three categories may be attributed to the variety of characteristics expressed among patients facing the same clinical endpoint.

Ovarian insufficiency constitutes a poorly understood entity (Giannelou et al., 2020). Adding a further level of complexity, established management options are limited. Hence, ART clinicians follow a common line of approach treating infertility caused by ovarian insufficiency, when in fact they are treating patients significantly heterogenic. In light of the lack of consensus or universally accepted protocols on treatment of ovarian insufficiency, fertility specialists are left challenged. This may in turn lead to the risk of enlisting empirical approaches. Following on this rationale, it is apparent that ovarian insufficiency patients certainly require thorough and effective profiling into subcategories. Biomarkers have emerged as promising means to that end. The question is “is enough genetic and molecular data available, and if so, are they aptly employed in the service of precision medicine when understanding and treating ovarian insufficiency?” Along these lines of exploring the optimal biomarkers for molecular profiling, micro-RNAs (miRNAs) appear as the ideal candidates. This may be true due to a variety of reasons, for instance their long half-life span (Pettit et al., 2018). miRNAs are part of the complex posttranscriptional mechanisms that cells recruit in order to regulate gene expression. Regulation of gene expression constitutes a multifaceted process encompassing several factors. miRNAs are short non-coding RNA molecules encompassing 18–24 nt (Zhang et al., 2019). miRNA genes are either located in various districts across chromosomes or convened in clusters along the same segment of a chromosome. These clusters may be perceived as a synergy targeting and regulating similar pathways (Tanzer and Stadler, 2004), fine-tuning cell and tissue functions (Maalouf et al., 2016). Interestingly, it has been proposed that the majority of mature miRNA transcripts contain miRNA response elements rendering miRNAs a crucial regulatory weapon participating in almost all biological pathways of physiology. A single miRNA may regulate and control more than one gene, and one gene may be under the strict monitoring and control of multiple miRNAs (Brennecke et al., 2005; Lewis et al., 2005; Lim et al., 2005; Friedman et al., 2009). Furthermore, miRNAs are incorporated into a silencing complex and therefore are able to interact with specific mRNAs resulting in their destabilization or/and repression (Friedman et al., 2009). Cell proliferation, differentiation, apoptosis, hormone synthesis, and secretion are believed to be regulated by miRNAs (Imbar and Eisenberg, 2014). The expression levels of miRNAs in the reproductive tissues seem to be related to female fertility potential, as well as to embryo developmental capacity (Salas-Huetos et al., 2019). What is more, they may play a crucial role in the process of oocyte maturation and folliculogenesis (Imbar and Eisenberg, 2014). Inevitably, their potential role as biomarkers pertaining to infertility detection has also been proposed (Salas-Huetos et al., 2019). This is in accordance with wider studies that propose miRNAs as biomarkers aiming to facilitate diagnosis of patients in the field of medicine (Condrat et al., 2020).

In the present systematic review, we focus on three distinct categories of patients presenting with ovarian insufficiency while pursuing an infertility treatment. The research question raised was whether the employment of the novel tool of miRNAs could contribute to enable a clear distinction in the pathophysiological pathways involved in POR, POF/POI, and AMA patients. This is of added value seeing as the clinical phenotype of ovarian insufficiency shared by POR, POF/POI, and AMA patients overshadows their distinctive profiling identity from a molecular point view. Data employed herein are extracted from infertile patients subjected to *in vitro* fertilization (IVF) cycles. It is IVF treatment that enables the investigation of tissue-specific samples, subsequently allowing profiling of these patients from a reproductive perspective. Employing the foolproof approach of a systematic review analysis, we aim to showcase a distinct profile for each category from the perspective of the miRNAs involved. The respective targeted genes and corresponding regulated pathways are recruited in order to explore the level of similarity within each category. Further to that, potential weaknesses and discrepancies among published articles thus far may be highlighted. This will in turn provide the scientific community with incentive and guidance to proceed employing all available tools toward the molecular identification of patients with ovarian insufficiency. The unique molecular mapping provided may facilitate a more advanced approach in diagnosing and individually managing these cases. This systematic research constitutes the first attempt in literature to investigate the three distinct expressions of ovarian insufficiency under their respective molecular spectrum and concur on the extent of their discrepancies and similarities. The systematic investigation of the literature employed presented valuable data that served as a platform for a subsequent advanced analysis. Based on the detected miRNAs, the respective affected pathways were revealed employing a strategy of bioinformatics depicting the compromised molecular aspects of each pathophysiology examined.

## Materials and Methods

### Literature Search Strategy

A systematic computerized search was conducted in Pubmed/Medline, Embase, and Cochrane Central databases on the 11th of February 2020 and was updated on the 13th of October 2020. In order to identify original publications on miRNAs detected in the follicular fluid from patients with poor ovarian response, premature ovarian insufficiency, and advanced maternal age, an appropriate search strategy was designed. The search strategy included the following terms and their combinations: “small non-coding RNA;” “micro RNA;” “ovarian insufficiency;” “ovarian failure;” “premature ovarian failure;” “premature ovarian insufficiency;” “primary ovarian insufficiency;” “poor ovarian reserve;” “poor ovarian response;” “diminished ovarian reserve;” “premature menopause.” The original search yielded 152 studies from all included databases. Following removal of duplicate studies (*n* = 7), a primary study selection for detecting relevant articles was performed based on title and abstract screening as depicted in the flowchart of Preferred Reporting Items for Systematic Reviews and Meta-analysis (PRISMA) (Supplementary Figure 1). Selection of the relevant articles was performed independently by three authors. Disagreements and discrepancies among the selected articles were resolved by an arbitration mediated by the senior authors. Additional eligible studies were identified by forward and backward citation mining performed on the selected relevant publications. Full-length manuscripts published in English in peer-reviewed journals meeting the strict criteria for defining ovarian insufficiency were eligible for inclusion, resulting to a total of 5 studies.

### Inclusion and Exclusion Criteria

The present systematic review considered for inclusion only miRNAs detected in follicular fluid samples, including granulosa cells accumulated during stimulated IVF/ICSI cycles of patients with poor ovarian response, premature ovarian insufficiency, or being of advanced maternal age. Poor ovarian response classification was defined according to the Bologna criteria. Patients should be fulfilling at least two out of the three following criteria including: advanced maternal age (≥40 years old), previous POR described as previous IVF attempts resulting to a yield of less than three oocytes retrieved—or an abnormal ovarian reserve test including an antral follicle count (AFC) of less than five follicles—or anti-Müllerian hormone (AMH) levels less than 1.1 ng/ml. POF/POI was defined as the loss of ovarian activity prior to the age of 40, manifested by amenorrhea or oligomenorrhea, raised gonadotropins, and low estradiol levels. At this point, it should be emphasized that POF and POI are two terms employed to describe the same condition and are considered synonymous and may be used interchangeably (Torrealday et al., 2017). AMA was defined as patients over the age of 35 at the time of the IVF/ICSI cycle with an AMH < 5 pmol/L. Publications referring to long non-coding RNA, miRNA polymorphisms, or miRNAs in the circulating blood were excluded. Retrospective studies and case reports were not considered eligible for inclusion.

### Functional Analysis of miRNA Targets

Experimentally supported and *in silico* predicted miRNA–target pairs were retrieved from DIANA-TarBase (Karagkouni et al., 2018) repository and microT-CDS (Reczko et al., 2012; Paraskevopoulou et al., 2013), respectively. Experimentally supported miRNA interactions, defined in the cervix, uterus/endometrium, ovary, and ureter tissues, were retained, while a 0.9 cutoff threshold was applied in the predicted miRNA–gene pairs. Gene expression values were obtained from The Genotype-Tissue Expression (GTEx) (Yizhak et al., 2019) repository, to infer expressed miRNA targeted genes in ovary tissue. Processed RNA-Seq data from 180 non-diseased individuals were retrieved, and the mean Transcripts Per Million (TPM) values per gene were estimated. Genes with TPM > 1 were retained. Genes and miRNAs annotation were derived from Ensembl (Cunningham et al., 2019) and miRbase (Kozomara et al., 2019), respectively. The final set is composed of 4,332 experimentally supported and 15,245 predicted miRNA target pairs. Gene-set enrichment analysis of miRNA targets was performed for each category separately (POR, AMA, and POF), to define significant pathways according to the Kyoto Encyclopedia of Genes and Genomes (KEGG) (Kanehisa, 2019; Kanehisa et al., 2019) database and the biological implication of genes according to Gene Ontology (GO) (Ashburner et al., 2000; The Gene Ontology Consortium, 2019). The Gene Ontology Resource: 20 years and still GOing strong, 2019 annotation. Fisher’s exact test was realized with the use of R package limma (Ritchie et al., 2015), and a 0.01 *p*-Value threshold was applied.

## Results

### Poor Ovarian Response

#### Background Information on POR

Poor ovarian response is a term employed to describe the ovary’s inability to adequately respond to controlled ovarian stimulation protocols during the initiation of an IVF cycle. It has been estimated that approximately 9-4% of women subjected to an IVF cycle may fail to respond to stimulation protocol (Ubaldi et al., 2014). Clinicians managing patients with POR are encountered by a series of obstacles stemming from the limited number of oocytes initially yielded during the oocyte retrieval procedure. The low number of available oocytes will subsequently prompt a cascade of poor prognosis events including deficit of available fertilized oocytes and embryos, as well as practical limitations in efficiently selecting an optimal embryo to employ in the embryo transfer procedure, leading to lower pregnancy rate (Ubaldi et al., 2014).

Attempting to diagnose a patient with POR, several points of consideration should be acknowledged. Certain indications should be employed in clinical practice in order to guide clinicians toward a timely, efficient diagnosis and management. Distinguishing between what may serve as a criterion rather as an indication of poor ovarian response has been repeatedly attempted since the discrepancies among published studies was striking. It is these discrepancies that cast a shadow of doubt even on robust statements concerning how these patients should be clinically managed. To highlight the fact that researchers are far from being on the same page, when Polyzos and Devroey (2011) designed and conducted a systematic review on the treatment of poor responders, 47 randomized controlled trials were initially included, among which 41 different definitions of poor responder patients were implemented.

Lacking concrete criteria and a common consensus for diagnosis constituted a huge backpedal for research in the field of treating and managing patients of poor ovarian reserve. Despite expecting this controversy to be resolved by the Bologna criteria, they have been criticized due to the heterogeneity regarding patients’ age, oocyte competence, and risk factors entailed (Vaiarelli et al., 2018). Yet, they are considered the most inclusive and collected attempt so far in literature (Ferraretti and Gianaroli, 2014). The comparison of any proposed clinical management evaluated on the grounds of safety and efficiency is unfeasible when discrepancies concerning criteria employed for selecting study population exist to such an extent. These ambiguous diagnostic criteria settlements and vague fundamental designing discrepancies among published studies fuel clinicians with uncertainty when diagnosing POR patients.

The impact of lacking strict criteria for diagnosing POR patients extends to a less than optimal management and treatment (Ubaldi et al., 2014). Despite the vast heterogenicity among patients, modifications in the hormonal stimulation protocol seem to serve as panacea in managing this pathology fully demonstrating the lack of tailored treatment options. It becomes evident that in terms of diagnosis and management, no conclusive line of approach has been proposed for this group of patients.

### Results of Systematic Review Along With miRNA and Affected Pathway Presentation on POR

Following literature screening, two prospective studies employing Bologna criteria to define POR patients were eligible for inclusion (Table 1). Aiming to identify unique miRNA patterns and their potential association with the biological mechanisms of POR, Zhang K. et al. (2017) studied the follicular fluid of 45 patients dividing them into two categories, one of young patients ranging from 25 to 35 years old (15/45), and one of patients over the age of 40, categorized as old (15/45). Fifteen patients with normal AFC undergoing IVF due to male or tubal factor infertility were included in the control group. Following miRNA sequencing on follicular fluid samples, a differential miRNA expression was observed between the three groups. As documented in Table 2, **86** miRNAs were identified in total. At this point, it should be highlighted that the categorization of findings presented as upregulated and downregulated miRNAs served the purpose of portraying our data in a comprehensive fashion. This study highlighted the differential expression of miR-15a-5p, miR-483-5p, miR-199a-5p, and miR-885-5p in POR patients compared to the control group. Following validation, miR-15a-5p and miR-483-5p were confirmed. The target gene of miR-15a-5p, *BCL2*, was detected significantly downregulated, whereas *BAD* was upregulated in young POR patients. In regard to miR-483-5p, IGF2 is considered its host gene and was detected downregulated in young POR patients. Following *in vitro* culture experiments and Western blot results, miR-15a-5p was concluded as playing a role in the pathogenesis of POR in young patients by regulating oocytes, granulosa cell proliferation, and apoptosis. When comparing old and young patients, no commonly expressed miRNAs were observed suggesting the different pathogenetic mechanisms entailed in each age group of POR patients. GO pathway and network enrichment analysis indicated the connection between the miR-15a-5p and PI3K-AKT-mTOR signaling pathways, apoptosis, GnRH signaling pathway, and estrogen signaling pathway. The second study included in the present systematic review was performed in 2019 by Luo et al. (2019). Poor ovarian reserve patients were included aiming to determine their miRNA profiling. Follicular aspiration was performed in 7 patients, and subsequent RNA extraction was conducted. HiSeq sequencing results were annotated and classified. miRBase (v21.0) was employed to identify known miRNAs and Mireap software to predict novel ones. Twenty conserved and 3 novel miRNAs were detected to be upregulated in the POR group as presented in Table 2. The identified miR-23a-27a-24-2 cluster was highlighted and indicated as contributing to cell cycle and proliferation processes. It was further emphasized that the overexpression of miR-23a in granulosa cells could inhibit the expression of SIRT1 through the ERK pathway, while increasing the apoptotic events. Despite our efforts to contact the corresponding author in order to accumulate more data and categorize POR patients of this study in two groups based on their age, as performed by Zhang K. et al. (2017) no communication was established.

**TABLE 1:** An overview of the studies’ characteristics included in the systematic review.

Study	Category of patients	City/Country	Number of patients	Methodology	Pathways analysis
Diez-Fraile et al., 2014	AMA	Ghent, Belgium	21	miRNA extraction using a silica-gel-based membrane purification method. RT-qPCR was performed and further validation of the results was conducted.	miRNA predicted targets and KEGG pathway analysis was evaluated employing the Mirpath bioinformatics tool available on the DIANA LAB website.
Moreno et al., 2015	AMA	Valencia, Spain	30	Presence, proportion and quality of miRNAs were assessed with small RNA LabChips and BioAnalyzer. miRNAs Microarray analysis was performed to compare the miRNA profiles. RT-qPCR was employed to confirm the results from the pooled microarrays.	Diana-miRPath was employed to perform pathways analysis.
Chen et al., 2015	POF	Changsha, China	6	Total RNA extraction was performed with TRIzol reagent and was subsequently reverse transcribed using RevertAid First Strand cDNA synthesis kit.	TargetScan and micrRNA website were employed to detect targeted genes. No pathway analysis was further performed.
Luo et al., 2019	POR	Tianjin, China	7	RNA extraction was performed and integrity of RNA was assessed. High-throughput RNA sequencing was performed. HiSeq results were annotated and classified in GenBank database. miRbase was employed to identify known miRNAs, while novel miRNAs were identified with Mireap software.	miRanda and TargetScan software were implemented to predict target genes, which were then annotated with GO and KEGG database.
Zhang K. et al., 2017	POR	Shanghai, China	30	RNA extraction was performed and RNA quality was checked. RNA was then purified and further validated with electrophoresis. miRNA sequencing was performed. cDNA libraries were prepared. The EB-Seq algorithm was applied to identify differentially expressed genes. Verification of miRNA and mRNA expression was conducted	Targetscan was implemented to predict miRNA targets. GO analysis was performed. Pathways analysis according to KEGG database was utilized to identify significant pathways. Additional miRNA-mRNA network analysis was performed.

*miRNA, microRNA; RT-qPCR, Quantitative reverse transcription PCR; cDNA, complementary DNA; mRNA, messenger RNA; KEGG, Kyoto Encyclopedia of Genes and Genomes; GO, Gene Ontology.*

**TABLE 2:** Overview of upregulated and downregulated miRNAs in POR category.

upregulated miRNAs	downregulated miRNAs
hsa-miR-1197	hsa-miR-1180-3p
hsa-miR-133a-3p	hsa-miR-122-5p
hsa-miR-146a-5p	hsa-miR-1226-3p
hsa-miR-181a-2-3p	hsa-miR-1247-5p
hsa-miR-181a-3p	hsa-miR-1306-5p
hsa-miR-184	hsa-miR-133b
hsa-miR-23a-3p	hsa-miR-139-3p
hsa-miR-27a-3p	hsa-miR-1468-5p
hsa-miR-3614-5p	hsa-miR-1469
hsa-miR-378d	hsa-miR-23b-5p
hsa-miR-380-5p	hsa-miR-296-5p
hsa-miR-431-3p	hsa-miR-328-3p
hsa-miR-500a-3p	hsa-miR-3591-3p
hsa-miR-501-3p	hsa-miR-4279
hsa-miR-511-5p	hsa-miR-4286
hsa-miR-542-3p	hsa-miR-450b-3p
hsa-miR-654-5p	hsa-miR-483-3p
hsa-miR-15a-5p	hsa-miR-532-3p
hsa-miR-127-5p	hsa-miR-586
hsa-miR-199a-5p	hsa-miR-6087
hsa-miR-374b-5p	hsa-miR-636
hsa-miR-374c-3p	hsa-miR-885-5p
hsa-miR-376a-5p	hsa-miR-4422
hsa-miR-337-5p	hsa-miR-6510-3p
hsa-miR-122-5p	hsa-miR-8079
hsa-miR-193a-5p	hsa-miR-483-5p
hsa-miR-194-5p	hsa-miR-3664-3p
hsa-miR-199a-3p	hsa-miR-129-5p
hsa-miR-199b-3p	hsa-miR-135b-5p
hsa-miR-20b-5p	hsa-miR-32-3p
hsa-miR-214-3p	hsa-miR-376c-5p
hsa-miR-214-5p	hsa-let-7f-2-3p
hsa-miR-26b-5p	hsa-miR-4433a-3p
hsa-miR-3120-3p	hsa-miR-4433b-5p
hsa-miR-3120-5p	hsa-miR-4448
hsa-miR-3141	hsa-miR-450a-2-3p
hsa-miR-342-3p	hsa-miR-4700-5p
hsa-miR-3591-3p	hsa-miR-506-3p
hsa-miR-378a-3p	hsa-miR-508-5p
hsa-miR-3960	hsa-miR-509-3p
hsa-miR-497-3p	hsa-miR-513a-5p
hsa-miR-6795-5p	hsa-miR-513b-5p
hsa-miR-885-5p	hsa-miR-513c-5p
hsa-miR-452-5p	hsa-miR-655-3p
hsa-miR-106b-5p	hsa-miR-508-3p
hsa-miR-6722-3p	hsa-miR-493-5p
hsa-miR-343-3p*	hsa-miR-4751
hsa-miR-34-3p*	hsa-miR-326

*Not detected in miRBase (v21.0) that was employed to identify known miRNAs.

**FIGURE 1:**
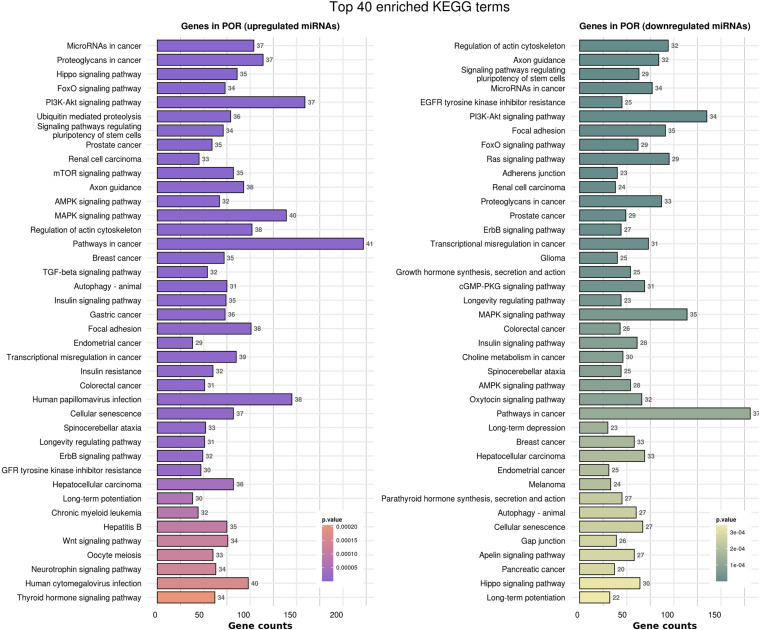
Functional significance of miRNA targets in the POR category. The top 40 significantly enriched KEGG pathways, containing targeted genes for the upregulated and downregulated set of miRNAs, are portrayed separately. The *X*-axis depicts the number of miRNA targeted genes enriching each term. Pathways are ranked according to the enrichment p-value (*p*-Value < 0.01, Fisher’s exact test). The number of miRNAs implicated in each pathway is shown at the end of each bar. Intensity legend shows the *p*-Value of each term.

**FIGURE 2:**
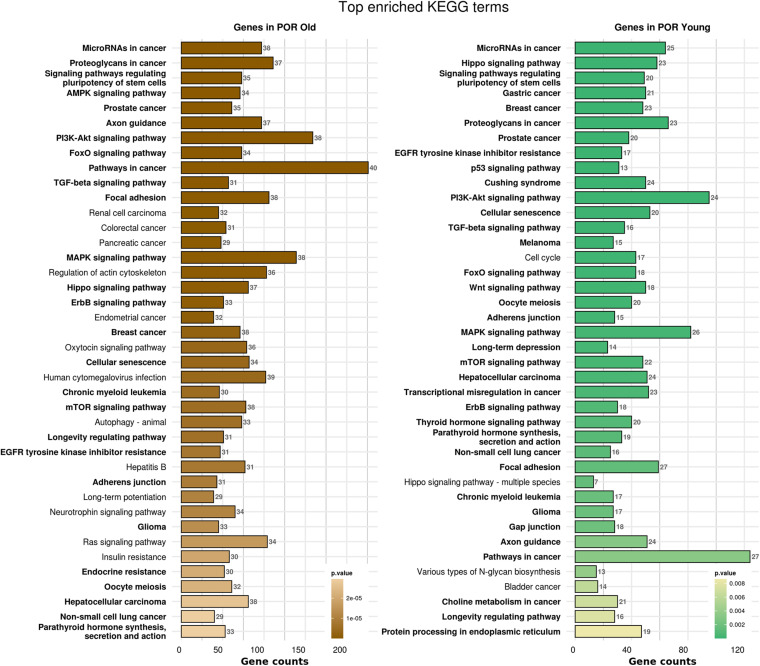
Functional significance of miRNA targets in POR old and POR young categories. The top significantly enriched KEGG pathways, containing targeted genes for the upregulated and downregulated set of miRNAs in each category, are portrayed. The X-axis depicts the number of miRNA targeted genes enriching each term. Pathways are ranked according to the enrichment p-value (*p*-Value < 0.01, Fisher’s exact test). The number of miRNAs implicated in each pathway is shown at the end of each bar. Intensity legend shows the p-value of each term. Common enriched pathways between the two categories are marked as bold.

**FIGURE 3:**
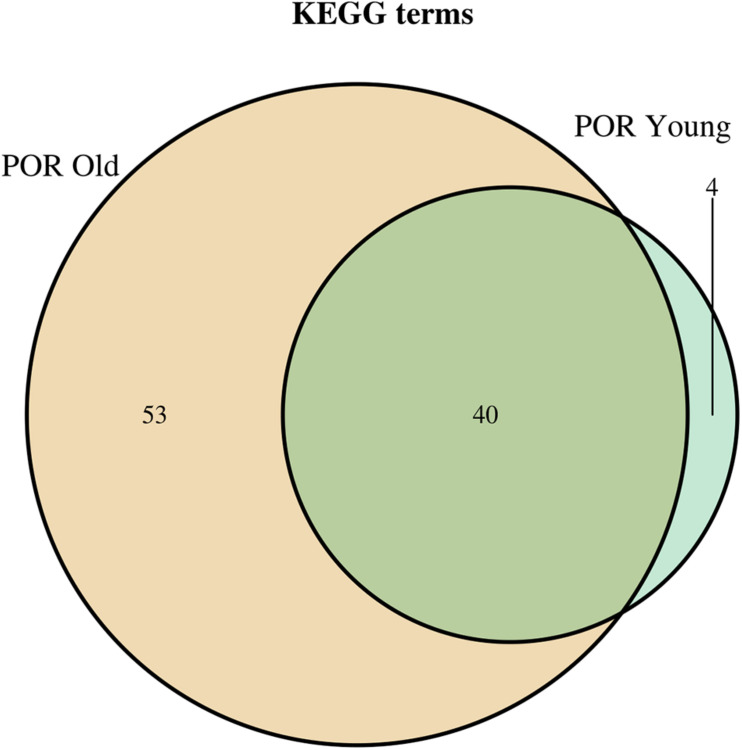
Venn diagram presenting POR old and POR young analyzed categories. The defined significantly enriched KEGG pathways, containing targeted genes for the upregulated and downregulated set of miRNAs, are compared.

In order to elucidate the molecular mechanisms implicated in POR pathophysiology, KEGG pathway analysis was conducted based on the detected miRNAs following data extraction. It should be noted that due to the complexity of the molecular pathways and the numerous participating genes, a conclusive assessment on the true impact a miRNA exerts on a pathway was not feasible. Based on the KEGG analysis of the reported upregulated and downregulated miRNAs, a list of numerous pathways was identified as presented in the Supplementary Tables 1, 2, respectively. The top 40 KEGG terms are depicted in Figure 1 concerning the top 40 pathways affected by upregulated and downregulated miRNAs in POR patients. The presentation of the top 40 pathways was based on their statistical significance and enrichment, containing targeted genes for the deregulated miRNAs of each category. Comparison of the profile of affected pathways between young and old POR patients was performed as in Figure 2, while Figure 3 portrays the commonly shared pathways between these two age groups of POR patients in an effort to assess whether the physiological mechanisms responsible for the onset of POR are the same irrespective of age. A literature investigation considering crucial pathways and their affiliation to impaired fertility potential in POR patients was conducted.

Crucial physiological pathways were affected highlighting the multifaceted and severely compromised mechanisms triggering a state of POR. Cell survival, cell growth, cell-cycle control, proliferation, differentiation, cellular senescence, and apoptosis constitute critical aspects indicating jeopardized cellular functions. Metabolic procedures, angiogenesis, steroidogenesis, ovarian aging, oocyte meiosis, and ovulation were also critical functions affected in POR patients according to KEGG analysis and subsequent literature investigation. Numerous signaling mechanisms and cellular interactions were affected including focal adhesion, adherens junctions, and gap junctions along with cellular motility including regulation of actin cytoskeleton and axon guidance. Interestingly, the endocrine system was similarly affected. Notably, the insulin signaling pathway, growth hormone synthesis, secretion, and action as well as parathyroid hormone were further jeopardized (Table 3).

**TABLE 3:** Overview of the affected physiological mechanisms in POR and AMA patients following KEGG analysis.

Affected pathways	POR	AMA
**Signaling pathways and signal transduction**		
PI3K-Akt signaling pathway	†	†
mTOR signaling pathway	†	†
TGF-β signaling pathway	†	
FoxO signaling pathway	†	
Wnt signaling pathway	†	
Hippo signaling pathway	†	
Ras signaling pathway	†	†
EGFR tyrosine kinase inhibitor resistance pathway	†	†
AMPK signaling pathway	†	†
MAPK pathway	†	†
p53 signaling pathway	†	†
N eurotrophins signaling pathway	†	†
ErbB(/EGFR) signaling pathway	†	†
JAK-STAT signaling pathway	†	†
**Cellular functions and interactions**		
Regulation of actin cytoskeleton	†	†
Oocyte meiosis	†	
Apoptosis	†	
Cellular senescence	†	†
Mitophagy		†
Gap junctions	†	
**Hormonal signaling**		
Growth hormone synthesis, secretion and action	†	†
**Protein Regulation**		
N-glycan biosynthesis	†	†
**Glucose Metabolism**	†	†

*^†^ This symbol indicates the category of patients a certain pathway is disregulated in.*

### Premature Ovarian Failure/Insufficiency

#### Background Information on POF-POI

This state of exhausted ovarian reserve in women under the age of 40 is described as POF/POI, and its prevalence is estimated at 1–2% (Coulam et al., 1986). The authors opt to employ thenceforth the term POF to refer to this pathology throughout the text as explained in the Materials and Methods respective section. The etiology of developing an ovarian insufficiency at such a young age is vague with iatrogenic, environmental, and autoimmune causes to have been reported (Huhtaniemi et al., 2018), including several factors such as infectious agents, metabolic disease, and autoimmune diseases and associated syndromes. Under these circumstances, it is the patients’ impaired ovarian reserve accompanied by the severe clinical issues that synergistically hinder the potential of achieving a pregnancy, while the risk of complications being substantially elevated.

Contrary to the poor responder category of patients who may remain undiagnosed until infertility issues arise that prompt them to seek treatment, POF is accompanied by severe symptoms and an overall compromised health condition copying menopause. Despite the long list of symptoms, the majority of which constitute indications of POF, strict criteria have also been proposed and established in clinical practice for diagnosing patients. Women under the age of 40, not reporting menstruation for 4–6 months, with FSH serum levels exceeding 40 mIU/L, and hypoestrogenism are considered POF (Shelling, 2010).

Treatment of POF patients is prescribed to either contribute to their fertility restoration or to ameliorate intensity of the symports they experience to subsequently enhance quality of life (Chapman et al., 2015). Hitherto, various strategies have been proposed, albeit none may be perceived as the holy grail of restoring fertility potential. The bulletproof approach in such cases is oocyte or embryo donation (Torrealday et al., 2017) with all that is entailed in such cases including patients’ reluctancy to embrace this option. In terms of mitigating symptoms of POF, hormonal replacement (HR) therapy is recommended similarly to menopausal women until the age of the normal menopause onset (Shelling, 2010). The question raised is to what extent is common ground shared between POF patients and older menopausal women regarding pathophysiology and respective molecular mechanisms. The fine line between the two pathophysiologies is the fact that in POF patients HR treatment aims to mimic the normal estrogen exposure these patients would normally experience, contrary to older menopausal patients for whom treatment aims to solely postpone menopause’s detrimental effects by extending women’s exposure to estrogens (Christin-Maitre, 2008). It is high time precision medicine highlighted further the differences between POF and menopause other than the apparent age difference being key in current diagnosis. However, apart from allowing comparisons, shedding light to the pathophysiology from a molecular perspective may enhance our hitherto impaired percipience concerning diagnosis and management of POF.

### Results of Systematic Review Along With miRNA and Affected Pathway Presentation on POF

In the present systematic review, one study has been evaluated as eligible for inclusion to investigate miRNA profiling in POF patients (Table 1). In Chen et al. (2015) investigated the role of miRNA-146a in the event of ovarian granulosa cell apoptosis where it was detected as upregulated in patients with POF. Isolation of granulosa cells from follicular fluid was performed and an ectopic expression of miR-146a was induced. RT-qPCR for miRNA and qPCR for mRNA were performed. miR-146a was detected as upregulated in patients with POF, which has been associated with cell apoptosis. miR-146a regulates the expression of IRAK1 and TRAF6 which have several implications in ovarian physiology, as well as in inflammatory responses. It was further highlighted that miR-146a participates in the apoptosis of granulosa cells through the TLR signaling pathway.

Kyoto Encyclopedia of Genes and Genomes pathways analysis was conducted in order to explore the role of miR-146a. Nonetheless, the fact that the analysis was based solely on a single miRNA resulted in the Herpes simplex virus 1 infection pathway being the sole affected pathway. No further evaluation could be performed. Thus, solely for the POF category, a Gene Ontology (GO) set enrichment analysis was performed to define the biological implication of miRNA-targeted genes. A list of the affected biological processes is presented in Supplementary Table 3.

### Advanced Maternal Age

#### Background Information on AMA

Childbearing over the age of 35 constitutes a unique category of patients referring to as the AMA group.

Aside from the underlying etiology, childbearing over the age of 35 has been associated with numerous pregnancy complications and severe clinical risks, namely, stillbirths and fetal growth restriction (Lean et al., 2017). Aging alone stands as a crucial parameter compromising fertility; any attempt to overcome it seems futile.

Despite the straightforward definition of AMA and the role of aging in the declined fecundity, management of these women pursuing treatment is challenging. AMA patients manifest poor response to ovarian stimulation protocols accompanied by poor-quality oocytes and subsequently embryos of low implantation potential (Yang et al., 2016; Nakagawa et al., 2019; Yin et al., 2019). This congregation of events will eventually result to high cycle cancelation rates, low pregnancy rate, and diminished live birth rates (Spandorfer et al., 1998; Younis et al., 2015). The lack of a common consensus perplexes the management process, while the huge heterogeneity of AMA patients adds another level of complexity for the clinician. This heterogeneity stems from the fact that the extent to which reproductive dynamics for AMA women are impaired strikingly varies. In contrast to the abovementioned types of ovarian insufficiency, AMA characteristics are linearly, directly, and unmistakably worsened with aging leading to the conclusion that “AMA from AMA” may differ considerably. In fact, women of ovarian insufficiency due to AMA are typically closer to and most commonly over the 40-year-old mark. The available therapeutic approaches for AMA patients over the mark of 40 years old pursuing fertility treatment are limited. Addressing the issue of optimal management is of paramount importance, especially in light of the upward trend of women pursuing fertility treatments at an advanced age.

#### Results of Systematic Review Along With miRNA and Affected Pathway Presentation on AMA

In the present systematic review, 2 articles were eligible for inclusion (Table 1). Diez-Fraile et al. (2014) investigated the differential miRNA profile in the follicular fluid of older women. Sixteen women participated in the study categorized in two groups based on their age. The group of young patients constituted of women under the age of 31, whereas the group of older participants included women older than 38 years old. RT-qPCR was performed following miRNA extraction for miRNA profiling and downstream analysis. For validation of results, a multiplex quantitative RT-PCR was conducted. Four miRNAs were differentially expressed among the age groups (Table 4). The role of miR-190b was highlighted in the regulation of heparan-sulfate proteoglycan expression, subsequently resulting in morphogenesis deregulation of the follicles in older women. What is more, alteration in carbohydrate metabolism was further documented in women of advanced age. It was concluded that the alteration in the expression of genes regulated by miR-190b could promote deregulation of glucose metabolism in the follicles of older women. miR-21-5 targets crucial genes of the p53 pathway, which plays the role of an anti-apoptotic factor, while target genes of miR-134 are participating in the apoptotic pathway of human granulosa cells. Therefore, the increased apoptotic events observed in the granulosa cells of AMA women could potentially be attributed to the downregulation of miR-21-5p and the enhanced expression of miR-134.

**TABLE 4:** Overview of miRNAs in AMA category.

upregulated miRNAs
hsa-miR-134-5p
hsa-miR-190b
hsa-miR-21-5p
hsa-miR-99b-3p
hsa-miR-424-5p

The second study by Moreno et al. (2015) explored the miRNA profiles of a cohort of patients based on their age. Following collection of follicular fluid, RNA extraction was performed and subsequent RT-qPCR analysis was completed. One differentially expressed miRNA was detected in patients of advanced age namely has-miR-424.

**FIGURE 4:**
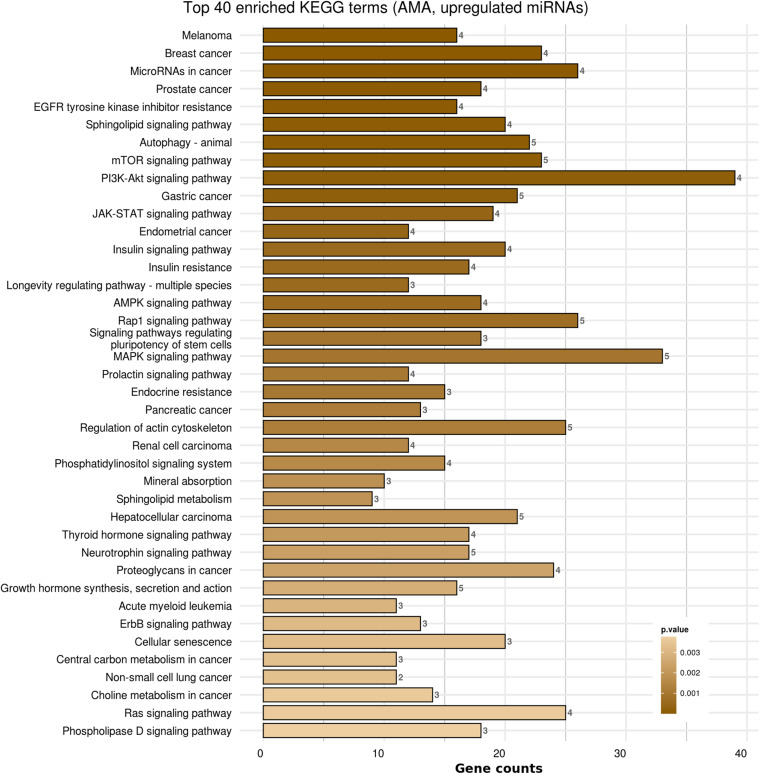
Functional significance of miRNA targets in AMA category. The top significantly enriched KEGG pathways, containing targeted genes for the upregulated set of miRNAs, are portrayed. The *X*-axis depicts the number of miRNA-targeted genes enriching each term. Pathways are ranked according to the enrichment *p*-Value (*p*-Value < 0.01, Fisher’s exact test). The number of miRNAs implicated in each pathway is shown at the end of each bar. Intensity legend shows the *p*-Value of each term.

**FIGURE 5:**
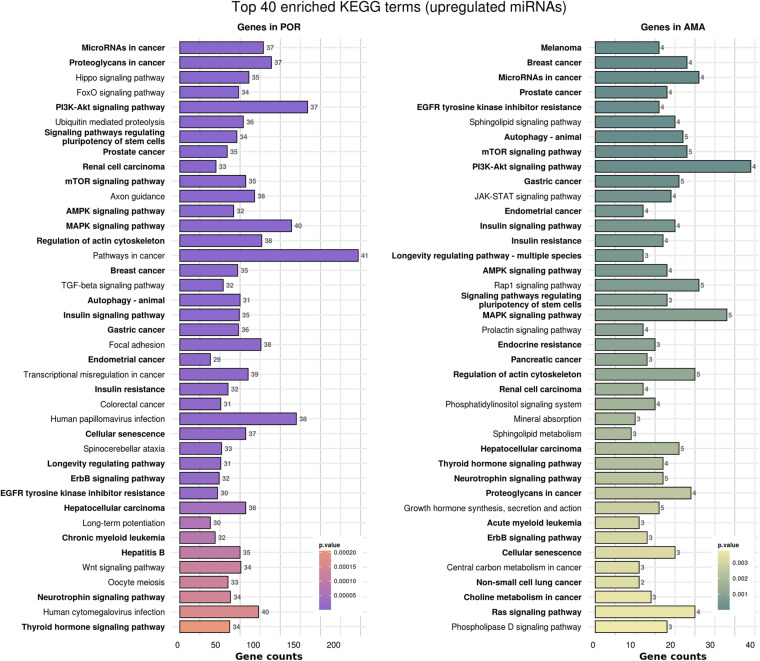
Functional significance of miRNA targets in POR and AMA categories. The top 40 significantly enriched KEGG pathways, containing targeted genes for the upregulated set of miRNAs in each category, are portrayed. The *X*-axis depicts the number of miRNA-targeted genes enriching each term. Pathways are ranked according to the enrichment *p*-Value (*p*-Value < 0.01, Fisher’s exact test). The number of miRNAs implicated in each pathway is shown at the end of each bar. Intensity legend shows the p-value of each term. Common enriched pathways between the two categories are marked as bold.

**FIGURE 6:**
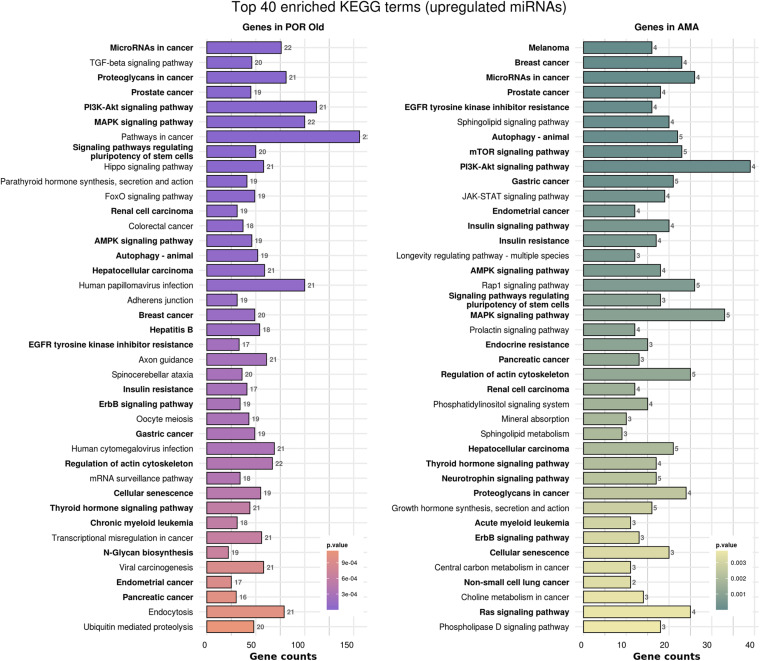
Functional significance of miRNA targets in POR old and AMA categories. The top 40 significantly enriched KEGG pathways, containing targeted genes for the upregulated set of miRNAs in each category, are portrayed. *X*-axis depicts the number of miRNA-targeted genes enriching each term. Pathways are ranked according to the enrichment *p*-Value (*p*-Value < 0.01, Fisher’s exact test). The number of miRNAs implicated in each pathway is shown at the end of each bar. Intensity legend shows the *p*-Value of each term. Common enriched pathways between the two categories are marked as bold.

**FIGURE 7:**
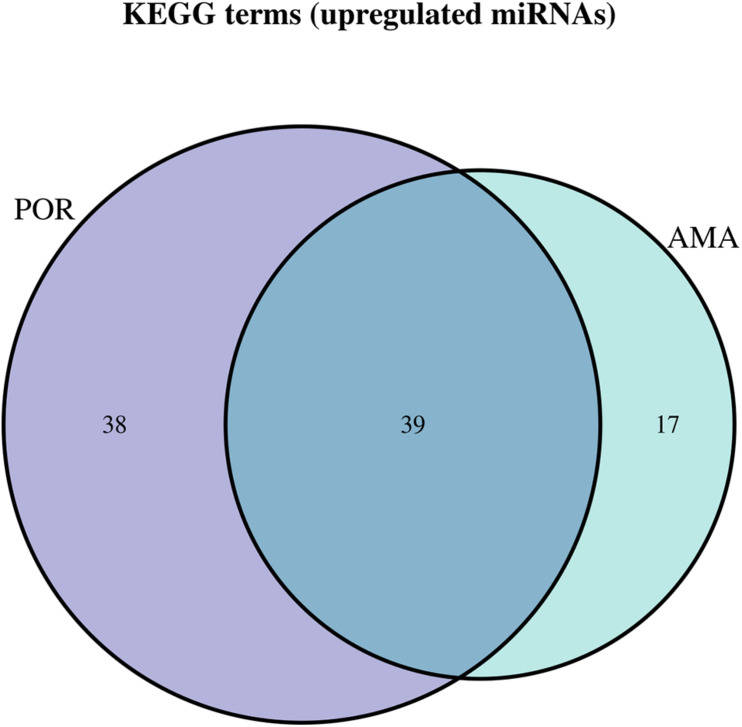
Venn diagram presenting POR and AMA analyzed categories. The defined significantly enriched KEGG pathways, containing targeted genes for the upregulated set of miRNAs, are compared.

**FIGURE 8:**
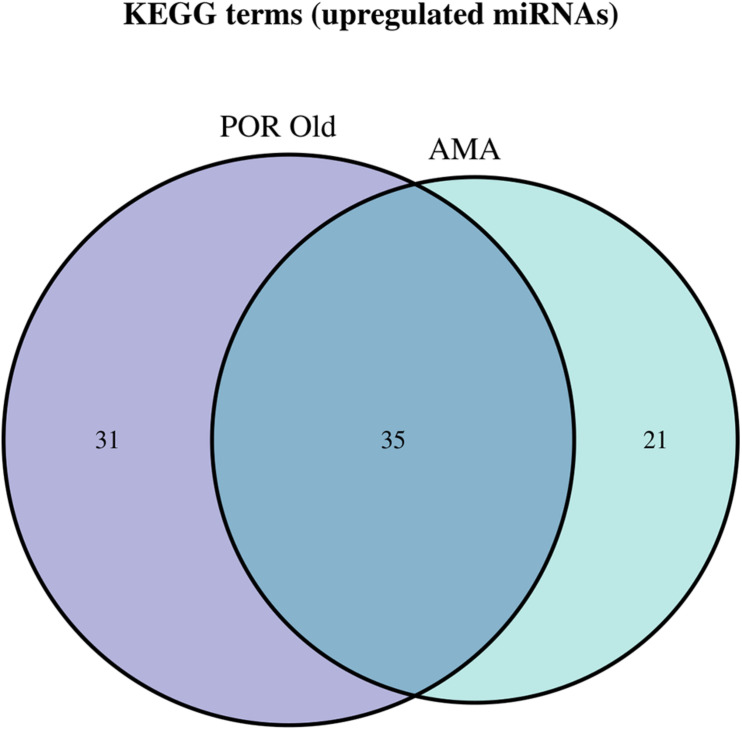
Venn diagram presenting POR old and AMA analyzed categories. The defined significantly enriched KEGG pathways, containing targeted genes for the upregulated set of miRNAs, are compared.

Kyoto Encyclopedia of Genes and Genomes pathways analysis was performed in order to provide insight into the molecular pathways affected in patients with AMA. Based on the KEGG analysis of the reported miRNAs, a list of numerous pathways was detected as presented in Supplementary Table 4. The top 40 KEGG terms are depicted in Figure 4 regarding the top 40 pathways affected by miRNAs in AMA patients. A comparison between AMA and POR patients as well as between AMA and old POR patients was attempted to demonstrate the potentially common ground these two pathophysiologies share (Figures 5–8). A literature investigation considering crucial pathways and their affiliation with impaired fertility potential in AMA patients was further conducted.

Energy balance, cell survival, cytoskeletal remodeling, cellular senescence, cell growth, cell cycle arrest, proliferation, differentiation, and angiogenesis constitute physiological processes regulated by affected pathways in AMA patients. Modulation of primordial follicle regulation, oocyte maturation, cumulus–oocyte complex expansion and ovulation, and granulosa cell steroidogenesis were also identified based on the extensive list of KEGG pathways and subsequent literature investigation. It should be highlighted that in AMA patients, dysregulation of metabolic procedures including mitochondrial reactive oxygen species (ROS) for stress resistance, mitochondrial biogenesis, mitophagy, and cellular metabolic balance were all indicated following literature search based on the detected pathways (Table 3). Interestingly, a limited number of affected pathways solely observed in AMA group of patients was recorded, such as the TNF-signaling pathway, which is a key component in ovarian development and function (Balchak and Marcinkiewicz, 1999).

### Analysis of Identified Affected Pathways Encountered in Ovarian Insufficiency

Micro-RNAs constitute a biomarker employed in an effort to reveal affected underlying mechanisms pertaining to the three expressions of ovarian insufficiency. However, the plethora of miRNAs detected calls for a “translation” as they lack the potential to provide data and report back to the practitioner. Based on the degree of relevance and affinity to the reproductive system, a selection process of the affected pathways was instigated. The selected pathways were further investigated in literature, and their connection to events of folliculogenesis, ovulation, and fertility was identified and further reported herein. Addressing the risk of potentially failing to identify crucial pathways that may affect future research in fertility, four authors examined independently the list of affected pathways and concurred on the crucial pathways that should be further examined and reviewed. Any discrepancies among the authors were resolved by the senior authors. The main principle of pathway selection in presenting the most relevant was to exclude pathways referring to general pathologies, or not strictly associated with the physiology of the reproductive system.

#### Signaling Pathways and Signal Transduction

The PI3K-Akt signaling pathway has been associated with intracellular signal transduction promoting physiological processes, namely, cell survival, metabolism, proliferation, angiogenesis, and cell growth (Hemmings and Restuccia, 2012). Based on our results, this pathway was compromised in POR and AMA patients. A cross talk between the PI3K/Akt pathway and the DNA damage response has been reported. This interaction regulates cell-cycle checkpoint initiation and DNA repair, while lack of phosphatase and tensin homolog (PTEN) causes genomic instability due to DNA double strand breakage (Brandmaier et al., 2017; Karimian et al., 2019). PTEN constitutes a tumor suppressor gene regulating the function of PIP3 which in turn inhibits signaling transduction by the PI3K/Akt pathway (Karimian et al., 2019). Surveillance mechanisms within oocytes are under investigation, and along these lines, transplantation of small fragments of the ovarian cortex treated with pharmacological inhibitors of PTEN has been proposed (Suzuki et al., 2015). Deletion of *Pten* has been proposed to activate the entire pool of primordial follicles and to provoke ovarian aging (Reddy et al., 2008, 2009). Interestingly, based on our results, *Pten* was affected by a detected miRNA and subsequently resulted to compromised PI3K-Akt signaling.

The mammalian target of rapamycin (mTOR) signaling pathway modulates various processes including protein synthesis, ribosome biogenesis, and autophagy (Guo and Yu, 2019). Hence, it regulates cellular functions, namely, metabolism, growth, proliferation, and differentiation, as a response to growth factors, nutrients, and energy status. TSC1–TSC2 constitutes pivotal multiprotein complexes involved in the mTOR pathway responsible for suppressing the activation of mTOR complex 1. Deletion of TSC1 or TSC2 leads to overactivation of the primordial follicle pool and hence depletion of the oocyte pool (Adhikari et al., 2009, 2010). Moreover, mTOR signaling has been related to crucial processes in female reproduction including folliculogenesis, oocyte meiotic maturation, ovarian somatic cell proliferation and steroidogenesis, puberty onset, ovarian aging, endometrium changes, and embryonic development (Guo and Yu, 2019). Enhanced activation of the mTOR pathway in oocytes and granulosa cells could potentially trigger primordial follicle activation and promote follicular development (McGinnis et al., 2013) which in turn determines ovarian reserve (Hirshfield, 1991). On the antipode, suppression of the mTOR signaling pathway impedes oocyte meiotic maturation (Guo et al., 2018). Following our analysis, the mTOR signaling pathway was found to be affected in POR and AMA patients, while both *Tsc1* and *Tsc2* were affected by a detected miRNA in the POR category.

Many of the signaling molecules involved in the dialog between the oocyte and the surrounding somatic cells belong to the transforming growth factor β (TGF-β) superfamily. Members of the TGF-β signaling pathway are involved in the control of proliferation and apoptosis of normal folliculogenesis and ovulation, while they promote estradiol release (Erickson, 2001; Yao G. et al., 2010). As expected, fertility issues could be triggered by defects in folliculogenesis. The normal growth of granulosa cells including proliferation and apoptosis seems to be dysregulated by the interaction of miRNA with the TGF-β signaling pathway (Chan et al., 2010; Zhang Q. et al., 2013; Nie et al., 2015; Tu et al., 2019). Interestingly, it has been reported that Smads, the signal transducers of TGF-β/GDF, could promote a specific set of miRNAs by assisting the cleavage reaction by Drosha. Our results confirmed that *Smad* were affected in POR patients by upregulated miRNAs.

The Forkhead box protein O (FOXO) family of transcription factors regulates the expression of genes in various crucial cellular physiological events including apoptosis, cell-cycle control, glucose metabolism, oxidative stress resistance, and longevity (Webb and Brunet, 2014). Their activity is under the influence of insulin and growth factor signaling which both act as inhibitors. Interestingly, pathway analysis reveals numerous interactions in which the FOXO signaling pathway is involved. The PI3K/AKT pathway is proposed to interact and trigger the FOXO regulatory pathways that subsequently affect crucial cellular functions (Matsuzaki et al., 2003). Based on our results, the FOXO signaling pathway was affected in POR patients.

Wnts constitute a large family of signaling molecules which have been associated with embryonic development (Logan and Nusse, 2004). What is more, it has been suggested that Wnt signaling plays an important role in female reproduction (Vainio et al., 1999; Savaris et al., 2011). Silencing the *Wnt4* in mice could provoke the loss of a large fraction of the reserve of oocytes. Subsequent studies have indicated that WNT4 expression is required during embryonic female gonadal and normal female fertility (Boyer et al., 2010). WNT4 regulates the function of granulosa cells and is required for antral follicle development since it regulates genes involved in several aspects of granulosa cell function (Vainio et al., 1999; Boyer et al., 2010), through the regulation of steroidogenesis (Lapointe and Boerboom, 2011). Following analysis, *Wnt4* was detected in the targeted genes in POR patients resulting to an affected Wnt signaling pathway.

The Hippo signaling pathway involved in mechanotransduction is considered one of the most crucial aspects of the cell’s ability to sense and respond to mechanical signals in order to regulate several cell functions including proliferation and apoptosis. The Hippo pathway is also involved in ovarian follicle development and specifically in the regulation of cell proliferation and apoptosis (Pan, 2007; Kawashima and Kawamura, 2018). Its disruption is related to *in vitro* activation of dormant follicles in cases of POR and early stage POF patients (Kawashima and Kawamura, 2018). A cross talk between Hippo, TGF-β and Wnt signaling pathways along with adherens junction is documented in genome databases. Based on our results, the affected Hippo pathway was detected in POR patients, along with the cluster of the aforementioned pathways that seem to contribute to common cellular functions.

The RAS signaling pathway regulates crucial events involved in fertility during ovulation and luteinization. Upon activation of the RAS pathway by LH, follicular granulosa cells are irreversibly committed to ovulation (Fan et al., 2012). The RAS GTPases transduce signals from extracellular growth factors and are considered molecular switches for several signaling pathways that regulate proliferation, apoptosis, cell cycle arrest, progression, cell survival, growth, and cytoskeletal remodeling (Vojtek and Der, 1998). A mechanism of action has been proposed based on observations from mutations in RAS which have been linked to POF cases. FSH holds the power to promote activation of RAS in granulosa cells; however, since POF patients are accompanied by elevated FSH levels, an overstimulation of granulosa cells will emerge that could provoke events of dysregulated RAS expression resulting to cell cycle arrest (Fan et al., 2012). Interestingly, the RAS signaling pathway was detected as affected in AMA and POR patients.

The activated epidermal growth factor (EGF)-like factors propagate the LH signal to granulosa and cumulus cells to induce ovulation, cumulus cell–oocyte (COC) complex expansion, oocyte maturation, and luteinization (Park et al., 2004; Hsieh et al., 2007). Studies with EGFR tyrosine kinase inhibitors and disruption of the EGF signaling pathway have demonstrated that this pathway is essential for the regulation of oocyte maturation, COC expansion and ovulation (Hsieh et al., 2007; Reizel et al., 2010). Its participation in events of reproduction, ovulation, fertilization, and embryo implantation has been further described (Schneider and Wolf, 2008). In AMA as well as in POR patients, the EGFR tyrosine kinase inhibitor resistance pathway was affected.

The adenosine monophosphate-activated protein kinase (AMPK) signaling pathway plays a role of a sensor of cellular energy status. This sensor allows cells to detect their energy capacity and adapt accordingly in order to survive and expand. It is activated by increased levels of cellular adenosine monophosphate (AMP): the adenosine triphosphate (ATP) ratio caused by metabolic stress that either interferes with ATP production or accelerates ATP consumption (McCallum et al., 2018). In case of a decrease in this ratio, AMPK initiates catabolic procedures while inhibiting anabolic pathways preserving ATP homeostasis. It has been proposed that due to its extensive expression in female reproductive tissues, it could be also implicated in reproductive functions such as granulosa cell steroidogenesis and nuclear oocyte maturation in several species. AMPK plays key function roles in regulating mitochondrial ROS for stress resistance and cellular metabolic balance (Rabinovitch et al., 2017), while promoting mitochondrial biogenesis (Herzig and Shaw, 2018). The AMPK signaling pathway was affected in AMA and POR patients.

The mitogen-activated protein kinase (MAPK) pathway connects extracellular signals to intracellular responses and facilitates events, namely, cell proliferation, inflammation, development, differentiation, and apoptosis (Zhang and Liu, 2002). The MAPK pathway is activated by the TGF-β receptors and is controlled by RAS activation. Its role has been primarily investigated in male infertility (Almog and Naor, 2010). Interestingly, based on our results, an affected MAPK pathway in AMA and POR patients was identified.

The tumor protein (p53) signaling pathway plays a pivotal role in the aging process. p53 activation is induced by a number of stress signals, including DNA damage, oxidative stress, and activated oncogenes. Furthermore, it affects physiological events, namely, cell cycle arrest, cellular senescence, and apoptosis. In human oocytes, p53 signaling pathway disruption could trigger genomic instability leading to aneuploidy (Guglielmino et al., 2011). Altered p53 expression has been documented in POR and AMA patients in literature (Krysko et al., 2008; Pacella et al., 2012), which was further confirmed by our analysis.

Neurotrophins are a family of polypeptide growth factors involved in promoting cell growth, differentiation, and survival of neural cells. Nonetheless, their role in the ovary has been investigated and interesting conclusions have been proposed. As suggested, neurotrophins participate in follicle assembly, primordial follicle activation, follicular growth, oocyte maturation, steroidogenesis, and ovulation. A strong indication of their interaction with the endocrine signaling system has been explored, and if dysregulated, ovarian reserve depletion should be anticipated (Chang et al., 2019). Both AMA and POR patients presented with the compromised neurotrophin signaling pathway.

The ErbB family of receptor tyrosine kinases participates in binding of extracellular growth factor ligands to intracellular signaling pathways, resulting in regulation of various biological responses, including proliferation, differentiation, cell motility, and survival (Ayuso-Sacido et al., 2010). The ErbB/EGFR signaling pathway was reported as affected in AMA and POR patients.

Janus kinase/signal transducers and activators of transcription (JAK-STAT) modulate the activation of primordial follicle regulation as well as follicle depletion through apoptosis. JAK1 and STAT3 are expressed in granulosa and oocytes of primordial follicles (Sutherland et al., 2018). Te JAK-STAT signaling pathway was identified in AMA and old POR patients.

### Pathways Involved in Cellular Functions and Interactions

Regulation of actin cytoskeleton was also reported in both AMA and POR. Asymmetric division of the oocyte attributed to actin dynamics plays a crucial role during fertilization and embryo development (Sun and Kim, 2013). Several miRNAs have been implicated in abnormal oocyte meiosis and spindle structure, but the precise mechanism is yet to be deciphered (Cui et al., 2013; Xiao et al., 2014). mTOR controls the actin cytoskeleton in many models, and, with regard to oocytes, it is required for asymmetric division in the mouse (Lee et al., 2012; Sun and Kim, 2013). Disrupting the function of both mTORC1 and mTORC2 complexes when the mTOR pathway is affected, spindle migration and asymmetric division are inhibited which has been described as actin-dependent since spindle migration is blocked when actin polymerization is inhibited (Yi and Li, 2012; Guo and Yu, 2019). In turn, asymmetric division depends on spindle migration. Based on our results, both mTOR and regulation of actin cytoskeleton pathways were affected by upregulated miRNAs in POR patients. Inhibition of mTOR may result in disrupted spindle migration, asymmetric division, and affected formation of actin cap (Lee et al., 2012).

Oocyte meiosis is a crucial process regulated intricately. Initially, the reduction in oocyte cyclic adenosine monophosphate (cAMP) levels is a prerequisite for the activation of cyclin-dependent kinase 1 (CDK1). It has been suggested that certain miRNAs inhibit the proliferation of pregranulosa and reduce the expression of CDKs and cyclins, thus eventually inhibiting the formation of primordial follicles (Zhang J. et al., 2013). Interestingly, our results indicated that CDK gene was targeted in POR patients indicating a compromised oocyte meiosis pathway.

As expected in cases of ovarian insufficiency, apoptotic events and apoptotic pathways are affected. miRNAs may either promote or repress apoptosis of granulosa cells (Tu et al., 2019). Accelerated rates of follicle activation and higher rates of atresia have been documented in POR (Nelson, 2009; Fan et al., 2019). An increase in cumulus cell apoptosis has been reported for AMA patients (Lim and Tsakok, 1997; Sadraie et al., 2000), albeit our analysis provided data on affected apoptotic pathways solely in POR patients.

The cellular senescence pathway is activated by cells in response to a stressful signal and describes the cell’s inability to divide following a growth stimulus (Velarde and Menon, 2016). Such stimuli may involve telomere shortening, oxidative stress, ionizing irradiation, and DNA damage. Following activation of senescence, huge metabolic and morphological modifications and adaptations occur (Ben-Porath and Weinberg, 2005). Cellular senescence is functionally associated with many biological processes including aging, tumor suppression, placental biology, embryonic development, and wound healing. Interestingly, oocytes deriving from women with IVF failures, recurrent miscarriages, or fragmented/aneuploid embryos tend to present with shorter telomeres (Treff et al., 2011; Mania et al., 2014). Based on our results, AMA and POR patients both presented with the compromised cellular senescence pathway.

Mitophagy refers to the removal of damaged mitochondria and constitutes a specific type of autophagy; a critical event for maintaining proper cellular functions (Ding and Yin, 2012). Alterations in mitochondrial biogenesis and mitophagy are important parameters to consider in women of advanced age. The mature oocyte contains numerous mitochondria in order for a sufficient number of mitochondria to sustain embryogenesis since mitochondrial replication in embryos initiates after implantation (Thundathil et al., 2005; Boudoures et al., 2017). It has been proposed that mitochondrial function is significantly impaired in cumulus cells of POR patients (Lin et al., 2017). Furthermore, an increase in the mitochondrial DNA content attributed to defective mitophagy or elevated stress has been described (May-Panloup et al., 2016; Woods et al., 2018). Mitophagy pathway has been identified as affected in the follicular fluid of AMA patients following our analysis.

Direct cell-to-cell communication via gap junctions plays a crucial role in pre-antral follicle growth. Gap junctions are abundant among granulosa cells surrounding oocyte (Eppig et al., 1996). Their importance lies on the fact that during folliculogenesis, amino acids, nucleotides, and metabolites are some of the pivotal molecules transferred through gap junctions (Eppig, 1991). Along these lines, signaling molecules regulate meiotic maturation travel through gap junctions. Subsequently, an arrest of the oocyte’s development is observed prior to accomplishing meiotic competence, highlighting the invaluable role of intercellular channels in oogenesis and ovulation (Simon et al., 1997; Ackert et al., 2001). Connexin43 (CX43) encoded by *Gja1* has been proposed to participate in follicular development, with evidence stating that CX43-deficient mouse follicles merely develop until the primary stage. According to our data, *Gja1* was detected as one of the targeted genes of miRNAs accumulated in POR patients, suggesting a compromised gap junction pathway involved in this pathology.

### Pathways Involved in Hormonal Signaling

Folliculogenesis and steroidogenesis are tightly regulated processes which involve gene expression, signaling pathways, and endocrine and paracrine factors (Imbar and Eisenberg, 2014). miRNAs function mainly on the granulosa cells to regulate steroidogenesis by controlling their growth, proliferation, and apoptosis. It has been stated that *in vitro* cultured human granulosa cells transfected with pre-miRNAs present either an inhibition of progesterone or estradiol release or some of the cells promote progesterone release (Sirotkin et al., 2014; Reza et al., 2019). miRNAs are involved not only in estradiol synthesis but also in estradiol release by granulosa cells (Yin et al., 2012). Furthermore, the expression of miRNAs is controlled by the level and composition of steroids in the follicle (Fiedler et al., 2008; Yao et al., 2009; Yao N. et al., 2010; Donadeu et al., 2016). Several studies have demonstrated that certain miRNAs either repress or promote hormone production via several pathways, namely, granulosa cell apoptosis, proliferation, TGF-β, Wnt signaling, cAMP signaling, FOXO, or Ras or by binding directly to the UTR of the aromatase coding sequence (Sirotkin et al., 2010; Xu et al., 2011; Yin et al., 2012; Dai et al., 2013; Sang et al., 2013; Wang L. et al., 2016). The growth hormone (GH) is considered a pivotal factor in ovarian angiogenesis and its role in regulation of ovulation and fertility has been indicated (Devesa and Caicedo, 2019). The fact that GH and gonadotropins have been implemented in the treatment of POR for decades highlights its invaluable contribution in ovarian physiology during all stages of ovarian development. Growth hormone synthesis, secretion, and action pathway were included among the list of compromised pathways in AMA and POR patients.

### Pathways Involved in Protein Regulation

Posttranslational addition of oligosaccharide residues has been described as a modification secreted protein may endure that alters their folding, trafficking, stability, hydrolytic properties, and hence their functions. One of the five major classes of glycans, N-glycans are components of N-glycoproteins that have pivotal roles in cell migration, angiogenesis, and cell–cell adhesion (Kuo et al., 2011). It has also been indicated that they are involved in the regulation of oocyte maturation and ovulation by regulating fibrinolysis, blood coagulation, angiogenesis, extracellular matrix stabilization, cholesterol homeostasis, inflammation, and complement and antioxidant pathways in stimulated IVF/ICSI cycles (Lim et al., 2017; Patil et al., 2019). Additionally, heparan sulfate proteoglycans contribute to the patterning of oocyte signaling and cumulus cell function (Watson et al., 2012). It has been voiced that deletion of *Mgat4a* gene which participates in initiating the β(1,4)GlcNAc branch on N-glycans results in metabolic phenotype and diabetes (Ohtsubo et al., 2005). Interestingly, in both AMA and POR patients *Mgat4a* was one of the targeted genes that subsequently triggered an affected N-glycan biosynthesis pathway.

### Glucose Metabolism

Oocytes depend on cumulus cells to metabolize glucose, which is subsequently utilized by oocytes to produce the energy required for maturation (Su et al., 2009). Abnormal glucose metabolism in the follicles of ovarian-deficient patients has not been thoroughly investigated. Interestingly, insulin signaling has been associated with aging (Das and Arur, 2017). It has been reported that certain miRNAs may regulate insulin secretion and play a key role in regulating insulin resistance (Ling et al., 2009; Zhao et al., 2011; Sang et al., 2013). The insulin signaling pathway and insulin resistance were detected as affected in AMA and POR patients.

## Discussion

The “phenotype” of deteriorated ovarian function is a typical characteristic of patients presenting with POR, POF, and AMA in clinical practice. This systematic review focused on delineating whether ovarian insufficiency—which stands as the common denominator among the three pathophysiologies—could further reflect similarities on a molecular level of investigation. The cohort of patients diagnosed with ovarian insufficiency is vaguely defined and comprises of various classifications that present an overlap of characteristics. These vague lines for distinguishing these categories of patients are attributed to the vast heterogeneity of clinical characteristics they exhibit, perplexing their diagnosis and management in clinical routine practice. Investigating toward development of a novel tool that could enable profiling and facilitate a more accurate categorization of patients among the three pathologies of ovarian insufficiency constituted the fundamental driver of this investigation.

miRNAs have been introduced as a potential tool toward better comprehending underlying molecular mechanisms of pathological entities. Furthermore, engaging miRNAs as biomarkers may enable tailoring management according to a certain molecular profile of a patient as they constitute a unique expression of an individual’s molecular footprint. The present study aimed to identify miRNA profiles involved in ovarian insufficiency and subsequent infertility and provide data on the cross talk among miRNAs and their targeted genes, indicating the pathways affected. This was performed while attempting a further subclassification reporting on the three major categories of patients diagnosed with ovarian insufficiency while pursuing a pregnancy via assisted reproduction treatment. The documentation of all miRNAs detected in the ovarian tissue was further enriched by reporting on a list of all affected pathways and mechanisms associated with their performance and course of action. Studies employing follicular fluid were included as it consists of products of metabolism of the granulosa cells as well as the plasma which could cross the blood follicular barrier (Schweigert et al., 2006). Molecular analysis of the follicular fluid has the potential to provide important evidence for the pathophysiology of the ovarian niche which presents as compromised in ovarian insufficiency. The present study in its entity employs the platform of a systematic review approach to collect data on miRNAs as its basis to build further toward a bioinformatic investigation of miRNAs indicating the pathways affected, subsequently enabling a critical analysis for each of the three categories. The multiple levels of analysis entailed coupled by the investigation of not one but three pathologies account for the complexity of the results presented. This study constitutes an innovative and all-inclusive research attempt.

Hitherto, studies shedding light on the role of miRNAs have highlighted their multifaceted and complex role in pathological as well as physiological mechanisms (Zhang et al., 2019). However, interpreting results based on miRNA data is challenging and confusing and fails to provide an outlook of the mechanisms entailed. Translating our observations on the pathway level enables more thought-provoking conclusions and enriches the molecular profiles for each case. To some extent, profiling has been performed including pathways participating in events of follicular atresia, granulosa cell apoptosis, and steroidogenesis, either individually or in clusters as part of a complex network which was confirmed by the present analysis. Pathways involved in ovulation were identified in POR and AMA patients, namely, MAPK pathway, neurotrophin signaling pathway, FGFR signaling, EGF receptor signaling, TGF-β receptor signaling, Wnt signaling, insulin receptor signaling, and RAS signaling. Impaired ovulation constitutes the primary driver for these patients to seek fertility treatment; thus, ovulation mechanisms were expected to be compromised. What is more, hormonal imbalances are encountered in cases of ovarian insufficiency and may even propagate a detrimental decline in patients’ quality of life. Patients with ovarian insufficiency—such as POF—experience a chronic systematic condition accompanied by a myriad of symptoms due to the deprivation of crucial hormones, which in turn triggers both short- and long-term implications in patients’ well-being (Torrealday et al., 2017). Based on our analysis, RAS, PKB/AKT, and MAPK pathways were compromised in POR and AMA patients that have been proposed to participate in the FSH and LH complex network of molecular signaling. In addition, literature indicates that the GH receptor’s density is diminished in POR patients, whereas the GH receptor’s density appears to be improving with age only in normal responders (Regan et al., 2018). Our results demonstrated that GH synthesis, secretion, and action was compromised in both POR and AMA patients. Follicular regulation is a complex process that appeared to be further jeopardized based on our results. Activation as well as inactivation of primordial follicles involve various pathways including the JAK–STAT, FOXO, and PI3K/AKT among others (Holt et al., 2006; Reddy et al., 2008; Pelosi et al., 2013; Sutherland et al., 2018) which were confirmed by our findings. Interestingly, impaired processes involved in energy production, energy balance, and metabolism were revealed as evidenced by affected pathways namely AMPK signaling and mitophagy, suggesting that a decreased follicular developmental potential could manifest in patients diagnosed with POR and AMA. The expression of transcripts involved in cell cycle signaling pathways, spindle checkpoint regulation, DNA stability, oxidative stress, and ubiquitination was disrupted AMA patients, a finding consistent with previous studies (Grøndahl et al., 2010). Our results further demonstrate that pathways involved in cell cycle namely p53, cellular senescence, cGMP-PKG signaling pathway, pathways related to spindle stability namely regulation of actin cytoskeleton, and oxidative stress including p53, longevity regulating pathway, insulin resistance, and mitophagy are all deregulated by miRNAs detected in the follicular fluid of AMA patients, as indicated by studies. The physiological mechanisms involved have been aptly and collectively presented (Ubaldi et al., 2019) including gradual depletion of ovarian reserve, decrease of oocyte and embryo competence, and dysfunctional cohesins attributed to the physiological process of aging. Chromosome segregation, dysfunctional cohesins, shortening telomeres, and jeopardized mitochondrial metabolic activities have been indicated as parameters linked to the embryo’s competence (Cimadomo et al., 2018). Aging is proposed to impair mechanisms that may result to a lower energy production and balance, provoking a mitigated embryo developmental rate to the blastocyst stage, as well as a higher frequency of chromosome missegregation during maternal meiosis leading to a high increase in blastocyst aneuploidy rate—especially in women older than 35 (Franasiak et al., 2014; Capalbo et al., 2016).

In order to decipher the genetics along with the molecular mechanisms entailed in POF, it is important to acknowledge the phenomenon of follicular maturation considering the fixed number of primordial follicles a female is born with, along with the fact that only a few of them will develop and mature leading to release of a mature competent oocyte during ovulation. In a nutshell, the destiny of follicles involves either ovulation or atresia, with hormonal signals contributing to both pathways. Focusing further on the underlying genetic background of manifestation of POF, chromosomal abnormalities, single gene mutations, and polymorphisms have been suggested (Pu et al., 2014). However, in many cases, POF represents a complex pathology which could not be attributed to a single gene. On the contrary, a cross talk between multiple genetic defects and environmental features seem to contribute to the POF onset (Dixit et al., 2010). In our analysis, miR-146a was the sole miRNA included which is involved in the Herpes simplex virus 1 infection pathway by targeting numerous genes; non-etheless, its clinical significance in the manifestation of POF is doubtful. However, as evidenced in literature, apoptosis of ovarian granulosa cells is regulated by TLR signaling, via IRAK1 and TRAF6, which in turn affects the activity of NF-κB and IκBα, and the caspase cascade (Chen et al., 2015). Interestingly, TRAF6 and IRAK1 were detected in the list of affected genes by the miR-146a. Generally, miR-146a serves as a feedback suppressor for both the innate and adaptive immune responses by targeting factors (IRAK1, TRAF6, and STAT1) in the NF-κB and JAK-STAT signaling pathways (Boldin et al., 2011; Wang et al., 2013; Ho et al., 2014). Elevated expression of miR-146 has been linked to numerous autoimmune disorders, namely, rheumatoid arthritis, systemic lupus erythematosus, and inflammatory bowel disease (Li et al., 2018). Mitigated levels of miR-146-5p have been reported in various types of human cancer cells participating as a tumor suppressor (Li et al., 2016; Wang C. et al., 2016; Zhang X. et al., 2017; Si et al., 2018; Iacona and Lutz, 2019). Recent studies indicate that the combined genotypes and haplotypes of miR-146aC > G, miR-196a2T > C, and miR-499A > G are associated with increased susceptibility to POF; hence, the transcriptional deregulation of certain miRNAs could be involved in the onset of POF (Rah et al., 2013). Furthermore, certain miRNAs have been associated with POF based on studies in the plasma and ovarian tissues. miR-379-5p has been proposed to play a key role in the pathogenesis of POI as detected in biochemical POI patients. Its upregulation is linked to reduced granulosa cells’ proliferation and DNA impairment, providing evidence to support its role in the pathogenesis of POI (Dang et al., 2018). Reduced levels of miR-22-3p have been reported in blood samples from POF patients, which may reflect the diminished ovarian reserve whereas increased mir-23a has been reported to induce granulosa cell apoptosis (Yang et al., 2012; Dang et al., 2015). A series of up- and downregulated miRNAs have been reported in animal study models of POF, highlighting the significance of prostaglandin biosynthesis, response to hormonal stimulus and cell apoptosis pathways (Kuang et al., 2014). However, none of the abovementioned studies met the inclusion criteria to be considered eligible as discussed below. In an effort to shed light to the pathophysiological events that occur in POF and compensate on the lack of available data to annotate on, a GO terms analysis was performed solely in the POF group of patients.

Interestingly, no robust conclusions on the level of affected pathways were revealed by comparing the three pathophysiologies of ovarian insufficiency. As expected, a common genetic background shared between POF and AMA patients has been proposed by genome-wide studies. The initial design of the present study aimed to decipher and concur on the extent of similarities between the two. However, due to the limited number of miRNAs in the POF group and the limited studies included, comparison between the POF and AMA could not be performed at the posttranscriptional level of regulation. Similarities and common mechanisms were expected to be detected in all three pathologies as evidenced by our results. In terms of comparison between POR and AMA patients, interestingly a significant overlap of 45 common pathways was detected, despite the fact that POR constitutes a pathological disorder whereas AMA describes a physiological depletion of ovarian reserve due to aging. Probably, the common ground of ovarian insufficiency shared between the two suffices as an explanation on this matter. AMA covers a wide range of patients and can be further subdivided according to severity in relation to answering the question “how old it too old?”. For instance, albeit both 36- and 46-year-old patients are considered as AMA—according to definition—non-etheless 46-year-old patients may be more likely to also be POR. In other words, an AMA patient from another AMA patient may significantly differ despite sharing the same classification. The JAK-STAT signaling pathway and progesterone-mediated oocyte maturation were exclusively detected in the AMA category. AMA patients included in our study were over 37 years old within the normal range of AMH which was over the cutoff point corresponding to POR; hence, AMA patients were not poorly responding to stimulation in our included studies. Notably, the affected pathways by the miRNA target genes were significantly overlapping despite the impressive discrepancy between the two categories in regard to antral follicle count and AMH. Nonetheless, as Pelosi et al. (2015) aptly commented, since the beginning of a woman’s life, the ovary enters a trajectory course to its depletion and progressive loss of reserve, due to both aging and the monthly programmed recruitment of oocytes (Pelosi et al., 2015). Could the similarities observed between POR and AMA be attributed to this depletion dictated by age? To delineate this conundrum, a comparison between old POR patients and AMA patients was performed suggesting 43 common pathways, with progesterone-mediated oocyte maturation, TNF signaling pathway, and mitophagy pathway to be exclusively detected in AMA patients. Further studies need to explore this finding and concur on the molecular validity of adopting the same therapeutic management of HR in both categories of ovarian insufficiency.

A striking observation was that numerous detected pathways associated with crucial cellular functions and survival were repeatedly detected in all three distinct categories of patients. Proliferation and apoptosis of granulosa cells’ pathways—when affected—will instigate a series of deteriorating events that are expected to accompany the nadir of the follicular pool. Regarding POR, the initial comparison between young and old POR patients concluded on a limited overlap in their detected miRNAs indicating a discrepancy in biological processes manifesting ovarian insufficiency, potentially due to the substantial contribution of aging (Zhang K. et al., 2017). However, when performing a pathway analysis based on the aforementioned detected miRNAs, a considerable overlap of 43 pathways was detected, suggesting that the underlying molecular mechanisms related to clinical manifestations are similar. Nonetheless, no safe conclusions may be drawn based on miRNA profiling since there is a plethora of miRNAs, which may hold the potential to affect the same pathway. MiRNAs seem to constitute the go-between in capacitating regulation and surveillance of cellular pathways. What is more, based on our results, it could be extrapolated that younger POR patients tend to present with less affected pathways in comparison to older POR patients. This observation validates that age alone may stand as a detrimentally contributing factor in jeopardizing cellular function and behavior, which in turn explains how certain dysfunctions in molecular mechanisms may accumulate with age as anticipated.

There is a strong and inevitable connection between diagnosis and treatment. A more sophisticated diagnosis could enable further sub categorization and profiling of patients based on unique features on a molecular and genetic level. The importance of such features is intensified as clinical characteristics, and observations lack the potential to adequately distinguish patients diagnosed with the same pathology. In the timeline of events that induce tissue damage, impairment of molecular pathways always precedes the onset of phenotypically expressed dysfunctions in an individual. Thus, employing miRNAs as biomarkers to meticulously subcategorize patients based on their molecular footprint could provide a new perspective for individually diagnosing patients and subcategorizing them. Identifying unique patterns in ovarian insufficiency is imperative and dictates further research. To further this point, investigating treatments and lines of approaches under the prism of subgrouping patients may reveal their efficiency or the incompetence. Phenotypically expressed characteristics are clinically evaluated albeit the plethora of underlying mechanisms resulting in such an expression being commonly overlooked. This becomes important especially in light of the fact that any treatment may exert a different impact and be of variant performance even when opted for patients facing the same pathology. The perils of exposing patients to unsuitable medication or treatment, as well as the subsequent patients’ psychological and financial burden entailed in futile IVF attempts, showcase an alerting current practice for managing patients in the field of Reproduction.

Management of patients is proposed by recommendations not dictated by guidelines since robust data are required to conclude on the optimal line of approach. Is it the heterogeneity of patients that makes all efforts toward a consensus futile? And if so, could providing a means to profile patients address this highly heated topic? The extent of miRNAs’ implementation in clinical practice is not limited to its role in molecular profiling of the patients and in promoting personalized management. Regulators of miRNAs, their target genes and their effect in the related signaling pathways may further assist toward developing novel therapeutic approaches as they constitute key factors in regulation of cell function and activity (Li et al., 2015). Deciphering the pathways involved in the manifestation of a pathology could contribute toward implementing novel tools paving the way for further inventions of pharmacogenomic nature by either blocking or mimicking gene expression (Gilabert-Estelles et al., 2012). The therapeutic potential miRNAs hold is extraordinary and has been appositely proposed by several studies. This validates the enormous extent of applications miRNAs could exert while serving as biomarkers indicating pathological conditions.

Individualization of treatment in IVF is pursued to enhance successful outcome, to mitigate iatrogenic risks and secure the completion of each cycle (La Marca and Sunkara, 2014). Notably, to this day clinical practice appears to still be based on empirical approaches of therapeutic schemes in lack of robust data suggesting optimal therapeutic approach for each category of patients (La Marca and Sunkara, 2014). Most importantly, clinicians tend to opt for their line of strategy based on patients’ previous cycles in order to determine and adjust the parameters that may enable a future successful outcome. The scientific community should focus on addressing the need to engage on highly specialized tailor-made treatment options, being the holy grail of personalized medicine and defining quality of clinical practice. However, researchers seem to be—perhaps unnecessarily—exposed to the overwhelming maze of this confusing overload of information on the role of miRNAs that presents failing definition. There certainly is added value in incorporating the knowledge of miRNAs in well-designed clinical studies and ultimately in treatment. But how do we make good use of the promising tool provided through understanding the role of miRNAs? Bridging the gap between practitioners and tools of precision medicine requires connecting the dots and the pieces of available information. Therefore, an assessment of current literature regarding the implication of miRNAs in ovarian insufficiency is timely and essential. miRNAs could suppress or activate gene expression proving their perplexing role in the regulation of gene expression. This study cannot provide information pertaining to whether an identified miRNA suppresses or activates a precise pathway, only that it affects this specific pathway. A selection of studies investigating miRNAs in the ovarian tissue was conducted in order to focus our observations on their role in infertility. As Maalouf et al. (2016) aptly raised, elucidating the role of miRNA in a specific tissue does not unravel its role in other tissues, as miRNAs regulate differently on different cells and tissues. Despite the meticulous selection of studies in order to solely include data stemming from the ovarian tissue, certain unrelated pathways to fertility mechanisms were documented due to miRNAs multitargeted combinational effect in pathways. What is more, the current systematic review was designed abiding by a thorough research protocol in order to provide robust data and draw safe conclusions. On these grounds, strict inclusion criteria were established for the eligibility of the included studies. Following screening and citation mining, only a limited number of studies were eligible considerably restricting the pool of available data for processing. The list of miRNAs was restricted, and the three pathologies were not equally represented with regard to the number of studies corresponding to each category. This was particularly heightened in the case of the POF category of patients which included a single study on examining the levels of a specific miRNA. This major limitation of our study stems from the severe discrepancies we encountered in literature and which should be herein highlighted. Aside from the discrepancies in the detection method employed to define miRNAs, what was striking is that the definitions employed to diagnose patients lacked consistency and presented with a variety of approaches and categorization tools based on arbitrary criteria subject to each team’s experience on the matter of diagnosing patients. The alarming discrepancies in literature and the consequences this phenomenon exerts have been reflected in the fact that meta-analyses of the currently available trials on POR patients have been strongly discouraged since they may lead to the adoption of interventions of ambiguous value (Polyzos and Devroey, 2011). Albeit the wide range of definitions employed—failing to abide by the acknowledged criteria and guidelines—is a by-product of the challenging heterogeneity of patients with ovarian insufficiency, the fact of the matter remains. At any case, the remarkable inconsistency in literature prevented the authors from including further published articles in the systematic review part of this study. The lack of homogeneity and conformity with the established guidelines and recommendations should be voiced, as this very fact constitutes a huge backpedal on expanding scientific knowledge and proceeding with proper research methodology.

What is more, the tools employed during the functional analysis of this study are sensitive to the number of miRNAs included. However, the detected pathways that were identified were also confirmed by current literature highlighting that they indeed play a role in the pathophysiology of ovarian insufficiency. This finding strengthens the functional analysis results and buttresses our conclusions despite the fact that they relied on a limited number of miRNAs. Our results need to be validated in another external dataset, and further studies shall provide the final verdict. Nonetheless, this study may serve as the basis to build on future research on the subject. To conclude regarding the standing limitations of this study, in order to avoid investigating biomarkers following implementation of invasive procedures that could affect molecular activity such as the stimulation protocols employed during IVF/ICSI cycles, initially this study was designed to include studies implementing natural cycles; however, no adequate number of studies could be retrieved to perform this analysis.

The current systematic review highlights the need for more consistent future studies that will contribute to and promote excellence and evidence-based medicine. Further to that, navigating the overwhelming molecular data in an effort to delineate associations with clinical practice, it became evident that the missing link between the two fields is studies aspiring to provide guidance and convey a comprehensive message. This can be valuable for guiding future research that will ultimately report back to the practitioner strengthening patient management. For a sophisticated management and treatment of patients to be established, current practices in publication should focus further to highlighting the valuable information and encourage conduct of studies of an all-inclusive approach. On the matter of ovarian insufficiency and respective diagnosis and treatment, investigating and understanding the molecular background is a must that should non-etheless be coupled by studies providing a specific direction regarding the focus of future research.

In this systematic review, the knowledge gap on efficiently diagnosing and treating ovarian insufficiency served as an apposite example showcasing the requirement of employing a biomarker toward personalized approaches. This systematic review is the first attempt in literature to perform an analysis extending from miRNA profiling to the effect they exert on molecular pathways in the impairment of ovarian tissue in POR, POF, and AMA patients in the context of IVF treatment. Due to the fact that all three categories of patients were pursuing IVF treatment, their diagnosed ovarian insufficiency was established on the grounds of reduced fertility potential. The common denominator of the infertility treatments these patients were subjected to enabled the employed of follicular fluid—a tissue specific sample—that serves as an optimal candidate to perform analysis and profiling from a reproductive perspective. Nonetheless, results provided by the current analysis allow us to draw conclusions albeit to a limited extent. Employing miRNAs to distinguish between the three subcategories of ovarian insufficiency is a valid option worth exploring further in the future. miRNAs are promising tools that in the hand of researchers could serve as a powerful ally toward better understanding pathophysiology. Our analysis showcases the identity of molecular mechanisms and pathways associated with cellular processes such as signaling, apoptosis, and endocrine response commonly involved in the POF, POR, and AMA, respectively. What is of interest is that albeit common ground is shared regarding the affected molecular pathways, being the endpoint, it appears that it is the dysregulation of different miRNAs that leads through the shared affected pathways to the distinctive categories of patients POF, POR, and AMA, effectively distinguishing them. To conclude, the authors aim to highlight the complex molecular network that triggers the onset of ovarian insufficiency and set the tone for future studies to focus on exploring certain pathways that may play a key role in the manifestation of declined fecundity. Despite the fact that the comparison among the three pathophysiologies of POR, POF, and AMA could not be performed exhaustively due to the unavailability of eligible data, it was evident by our further analysis that all three substantially share common ground, as expected. What is more, a difference may be anticipated between miRNA profiling of an aging ovary caused by the depletion of ovarian reserve, in comparison to miRNA profiling corresponding to an underlying pathology. It is this biological basis that merits further investigation (Pelosi et al., 2015). Prior to investigating in-depth the unique molecular pattern in cases of ovarian insufficiency, the discrepancies in defining and categorizing patients should be removed from the equation. Researchers and clinicians should comply with the established guidelines and recommendations in order for their data to enrich current knowledge and promote optimal practice. Mapping the affected pathways for each of the three expressions of ovarian insufficiency and revealing similarities, overlaps, and differences on cellular and molecular levels brings to the spotlight novel data for further consideration.

## Data Availability Statement

The original contributions presented in the study are included in the article/Supplementary Material, further inquiries can be directed to the corresponding author/s.

## Author Contributions

AR, KP, and MS conceived and designed the project. AR, DN, KS, and PT performed the literature review. DK performed the bioinformatic analysis. AR, DN, SG, EM, AmP, and AV contributed to drafting and editing the manuscript. AgP, KP, and MS revised the manuscript. MK, AH, and MS contributed to the critical discussion and provided intellectual content. AH, KP, and MS supervised the study. All authors approved the final draft.

## Conflict of Interest

The authors declare that the research was conducted in the absence of any commercial or financial relationships that could be construed as a potential conflict of interest.

## Supplementary Material

Supplementary Figure 1 PRISMA flowchart presenting the search results.

Supplementary Table 1 An overview of the affected biological processes in POR category based on the extracted upregulated miRNAs following KEGG analysis.

Supplementary Table 2 An overview of the affected biological processes in POR category based on the extracted downregulated miRNAs following KEGG analysis.

Supplementary Table 3 An overview of the affected biological processes in POF category based on the extracted miRNA following GO analysis.
