# Supplementary figures and images for: Reporting on the Role of miRNAs and Affected Pathways on the Molecular Backbone of Ovarian Insufficiency: A Systematic Review and Critical Analysis Mapping of Future Research
Source: Front Cell Dev Biol. 2021 Jan 12;8:590106. doi: 10.3389/fcell.2020.590106 (PMC7835544; doi:10.3389/fcell.2020.590106)

**Supplementary Figure 1:** PRISMA flowchart regarding the search results

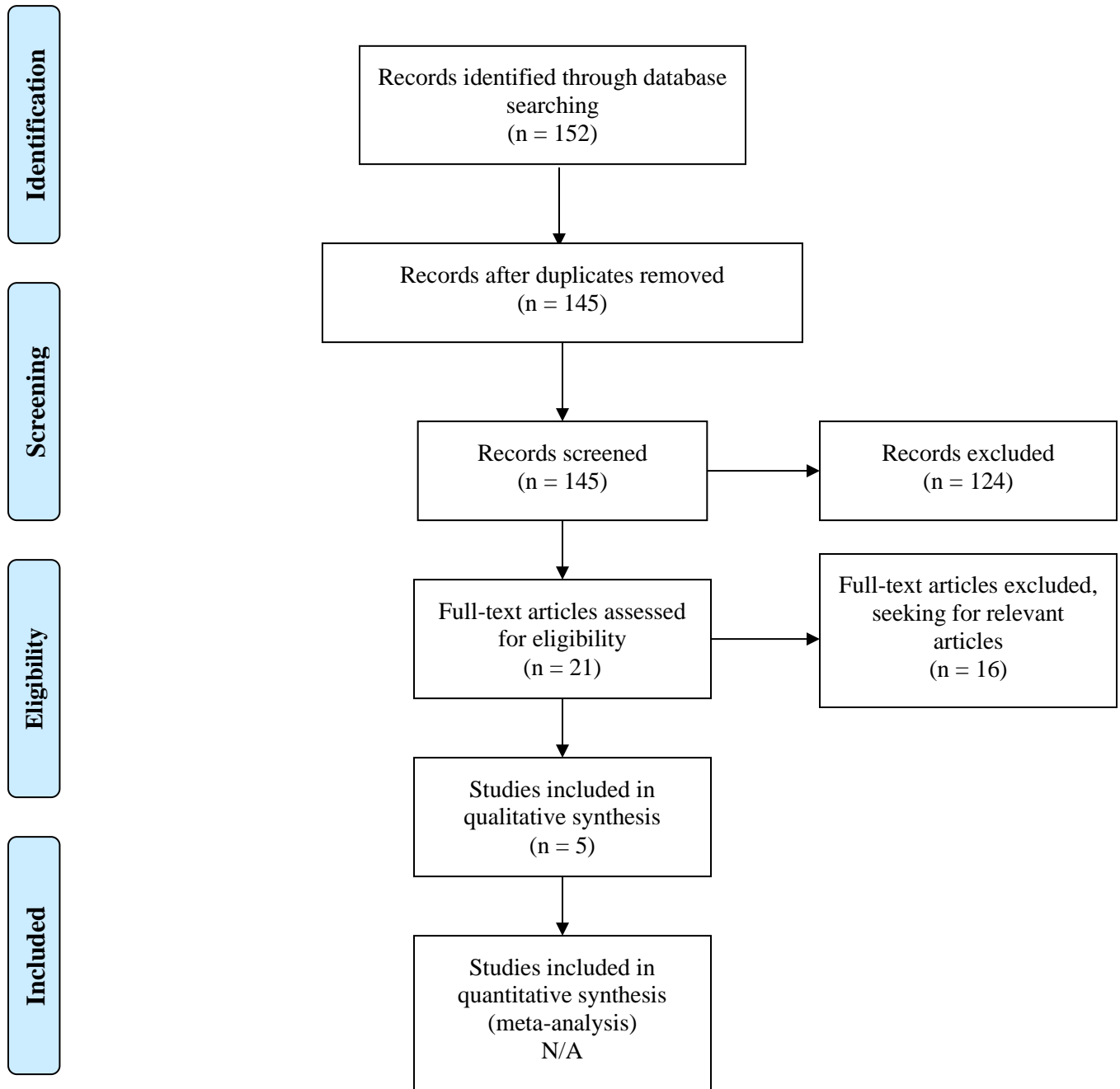

Supplement: Supplementary Figure 1 — PRISMA flowchart presenting the search results. [file Data_Sheet_5.PDF]
